# Stopping the clock on placental aging: time for physicians and scientists to work together

**DOI:** 10.1038/s41390-025-04269-6

**Published:** 2025-07-09

**Authors:** Karen K. Mestan

**Affiliations:** Department of Pediatrics, Division of Neonatology, University of California San Diego, La Jolla, CA USA

In a recent study published in *Pediatric Research*, Gerber et al. conducted a retrospective cohort analysis of 193 former extremely low gestational age newborns (ELGANs) to link placental epigenetic gestational age (eGA) acceleration at birth to adolescent systolic blood pressure at 15–18 years of age.^1^ This is the first study of its kind to test a novel hypothesis that alterations in placental function could program the developing kidney towards future cardiovascular disease. They describe the relationship between placental eGA acceleration (calculated from DNA methylation quantification using the Robust Placental Clock algorithm) and systolic blood pressure measurements obtained from adolescence clinic visits. The study aligns with the Developmental Origins of Health and Disease model that very early intrauterine exposures have lasting impact on adult cardiovascular health, conceptually through fetal programming during critical periods of organogenesis.

The fetal origins of disease concept has been used to validate placental findings at birth as a culprit (and/or a marker) of a wide range of health conditions, including cardiometabolic health (obesity),^2^ pulmonary outcomes (pulmonary hypertension and chronic lung disease)^3,4^ and long-term neurodevelopmental and behavioral health.^5^ An understudied area that Gerber and colleagues address is the potential lasting impact of placental dysfunction on the developing kidney. Knowing this association could help predict future risk of adolescent and adult cardiovascular disease related to chronic kidney disease—which remains a leading cause of death among both men and women.^6^

The classic histopathologic finding of abnormal placental aging is accelerated villous maturation (AVM), in which development of the villous trophoblast resembles structural changes seen at term gestation in births that have occurred preterm. Thus, villous maturation and placental aging are normal processes that occur throughout pregnancy. The causes of accelerated placental aging are poorly understood, but are likely associated with the indication for preterm birth, which itself is multifactorial. AVM is a sublesion of maternal vascular malperfusion (MVM) of the placental bed. The hallmark features of MVM include early abnormal trophoblast implantation, abnormal spiral artery remodeling in the decidua and downstream villous and vascular maldevelopment leading to compromised fetal blood flow, gas exchange and nutrient delivery.^7^ The onset, duration and extent of this compromise varies but is correlated with the degree of neonatal morbidity, with potential to negatively impact every organ system in the developing fetus—brain, heart, lung, intestine, and even the kidney.

Accelerated aging of the placenta is a known but re-emerging concept that has been linked to pregnancy conditions such as preeclampsia, fetal growth restriction and stillbirth. Assuming that placental eGA acceleration is a correlate of placental aging, the ELGAN investigators correlated calculations derived from the Robust Placental Clock to systolic blood pressure, taking into account pregnancy and other maternal conditions. They found that for every 1-week acceleration in eGA, adolescent males but not females had a significant increase in systolic blood pressure after adjustment for covariates. This association is novel and intriguing, but requires further investigation interrogating biologic plausibility, especially in this relatively small subsample of the ELGAN cohort. A major strength of the study is the characterization of a continuous measure of placental aging, albeit still only at birth, that can be assessed against continuous gestational age along the wide spectrum of early placental and human development. These findings could have important implications for how we approach prevention and management of early onset chronic hypertension in adolescence to address adult cardiovascular risks.

A unifying mechanism by which placental epigenetic aging might program the developing kidney towards chronic hypertension requires further investigation. Animal and in vitro models have contributed substantially to our understanding of how epigenetic priming of stress and inflammation negatively impact placental regulation of hormones to the fetus.^8,9^ These may serve as valuable models to further understand the sex-specific differences identified by Gerber et al. Other mechanisms linking placental aging and chronic vascular disease could be explained by anti-aging factors circulating at birth, such as circadian markers, epigenetic proteins, and anti-aging proteins such as Klotho.^10^ For example, the anti-aging protein Klotho is normally produced by placental trophoblast, with decreased expression in pregnancies complicated by preeclampsia and fetal growth restriction.^11^ Interestingly, Klotho serves as a key co-receptor for fibroblast growth factor-23 (FGF-23), which increases with progressive chronic kidney disease in the absence of Klotho.^12^ Thus, accelerated placental aging accompanied by epigenetic changes could alter Klotho expression, leading to downstream maldevelopment of the kidney through unbound FGF-23.

The authors speculate that the maternal exposome is primarily responsible for placental epigenetic aging, but without rigorous mechanistic studies placental epigenetic age serves largely as an imperfect predictor of future cardiovascular risk. Given that a single systolic blood pressure measurement obtained at an adolescent outpatient visit is the main outcome of the study, more compelling studies linking placental aging to kidney function 15 years later are needed in a larger cohort and with complementary biomarker studies. Moreover, without corresponding placental histology, it is challenging to understand how and to what extent accelerations in epigenetic age alter placental function.

In summary, an important message is that the mechanisms behind these placental-kidney associations require further investigation, before we can consider using such measures as the Robust Placental Clock as predictors of kidney disease and other chronic diseases. Identifying the mechanistic causes of placental aging should be a fundamental research priority, that has unfortunately fallen to the wayside. While the ELGAN cohort is a valuable resource for understanding the long-term outcomes of extremely preterm birth, it is important to also include the information that can further enhance our understanding of the epigenetic mechanisms—for example, the placental histologic findings such as AVM, MVM and contributions of acute and chronic inflammatory pathologies. Serial “hidden” clinical benchmarks and interim diagnoses (history of neonatal renal insufficiency/failure, use of nephrotoxic medications in the neonatal period), as well as blood and urinary biomarkers that could elucidate the biochemical and physiologic interactions between placental epigenetic age and postnatal mediators are needed. The study by Gerber and colleagues highlights that factors related to infant sex may be an important place to start. Going back to the bench with in vitro and in vivo models, coupled with state-of-the-art technologic advances in the -omics and AI to interrogate human placental and kidney function are much needed, and feasible more than ever. Understanding *how* maternal and infant environmental exposures induce epigenetic changes in the placenta--and how these exposures mediate the pathway between placental dysfunction and chronic disease—will help scientists and physicians develop and implement strategies to reduce long-term morbidity among our smallest and most vulnerable preterm patients.
